# Incident osteoarthritis and osteoarthritis-related joint replacement surgery in patients with ankylosing spondylitis: A secondary cohort analysis of a nationwide, population-based health claims database

**DOI:** 10.1371/journal.pone.0187594

**Published:** 2017-11-02

**Authors:** Ming-Chi Lu, Chien-Hsueh Tung, Chang-Chen Yang, Chun-Lung Wang, Kuang-Yung Huang, Malcolm Koo, Ning-Sheng Lai

**Affiliations:** 1 Division of Allergy, Immunology and Rheumatology, Dalin Tzu Chi Hospital, Buddhist Tzu Chi Medical Foundation, Dalin, Chiayi, Taiwan; 2 School of Medicine, Tzu Chi University, Hualien City, Taiwan; 3 Division of Orthopedics, Dalin Tzu Chi Hospital, Buddhist Tzu Chi Medical Foundation, Dalin, Chiayi, Taiwan; 4 Division of Pediatrics, Dalin Tzu Chi Hospital, Buddhist Tzu Chi Medical Foundation, Dalin, Chiayi, Taiwan; 5 Department of Medical Research, Dalin Tzu Chi Hospital, Buddhist Tzu Chi Medical Foundation, Dalin, Chiayi, Taiwan; 6 Dalla Lana School of Public Health, University of Toronto, Toronto, Ontario, Canada; Sahlgrenska Academy, Univeristy of Gothengurg, SWEDEN

## Abstract

**Background:**

Ankylosing spondylitis (AS) might be associated with an increased risk of secondary osteoarthritis. However, there is a lack of studies assessing its impact on osteoarthritis-related surgery. The aim of this secondary cohort study was to investigate the risk of symptomatic osteoarthritis and osteoarthritis-related surgery, including total hip replacement surgery (THRS) and total knee replacement surgery (TKRS) in patients with AS.

**Methods:**

Using the Taiwan’s National Health Insurance Research Database, we identified 3,462 patients with AS between 2000 and 2012. A comparison cohort was assembled consisting of five patients without AS, based on frequency matching for sex, 10-year age interval, and index year, for each patient with AS. Both groups were followed until diagnosis of the study outcomes or the end of the follow-up period.

**Results:**

Male patients with AS exhibited a significantly higher incidence of osteoarthritis (adjusted incidence rate ratio [IRR] 1.43; *P* < 0.001), THRS (adjusted IRR 12.59; *P <* 0.001), and TKRS (adjusted IRR 1.89; *P =* 0.036). Moreover, analyses stratified by age group (20–39 years versus 40–80 years) indicated a high IRR (adjusted IRR 27.66; *P <*0.001) for THRS among younger patients with AS.

**Conclusions:**

Male patients with AS had a significant higher risk of developing osteoarthritis, and receiving THRS and TKRS. Young patients with AS also showed a significant higher risk of receiving THRS.

## Introduction

Osteoarthritis is a chronic arthropathy characterized by structural degeneration of the joint, especially cartilage loss. It is a common cause of pain and lower extremity disability in the elderly population. The etiology of osteoarthritis is multifactorial with risk factors, including age, female sex, obesity, genetic predisposition, prior joint injury, and occupations involving heavy physical work load [1,2]. Osteoarthritis is usually classified as either primary (idiopathic) or secondary when it is caused by an underlying condition, such as injury to joints or a disease. Traditionally, osteoarthritis has been considered primarily as a degenerative condition of aging with a loss of cartilage resulting from an accumulation of damage over time [3]. However, recent research has suggested that inflammatory mediators released by cartilage, bone, and synovium play a pivotal role in the pathogenesis of osteoarthritis [4]. In addition to local inflammation that occurs within joint tissues, low-grade systemic inflammation associated with conditions such as obesity and metabolic syndrome could also contribute to an increased risk of secondary osteoarthritis [5,6]. Inflammatory factors, including cytokines, chemokines, adipokines, prostaglandins, and leukotrienes have been implicated in the pathogenesis of osteoarthritis [7].

Ankylosing spondylitis (AS) is a chronic, systemic, inflammatory autoimmune disease with a prevalence of 33.7 per 10,000 in Taiwan [8]. The disease typically occurs in young adults between the ages of 20 and 30 years with a male preponderance of 3:1 [9]. Although AS typically affects the axial skeleton and peripheral joints especially joints in lower extremities but nonarticular structures, such as the gastrointestinal tract, skin, eye, and aortic valve can also be involved [10]. We speculated that AS might increase the risk of secondary osteoarthritis. Therefore, the aim of this secondary cohort study was to explore the incidence of symptomatic osteoarthritis or osteoarthritis-related surgery, including total hip replacement surgery (THRS) and total knee replacement surgery (TKRS) in patients with AS using a nationwide, population-based health claims database in Taiwan.

## Methods

### Study design and data sources

**Fig 1:**
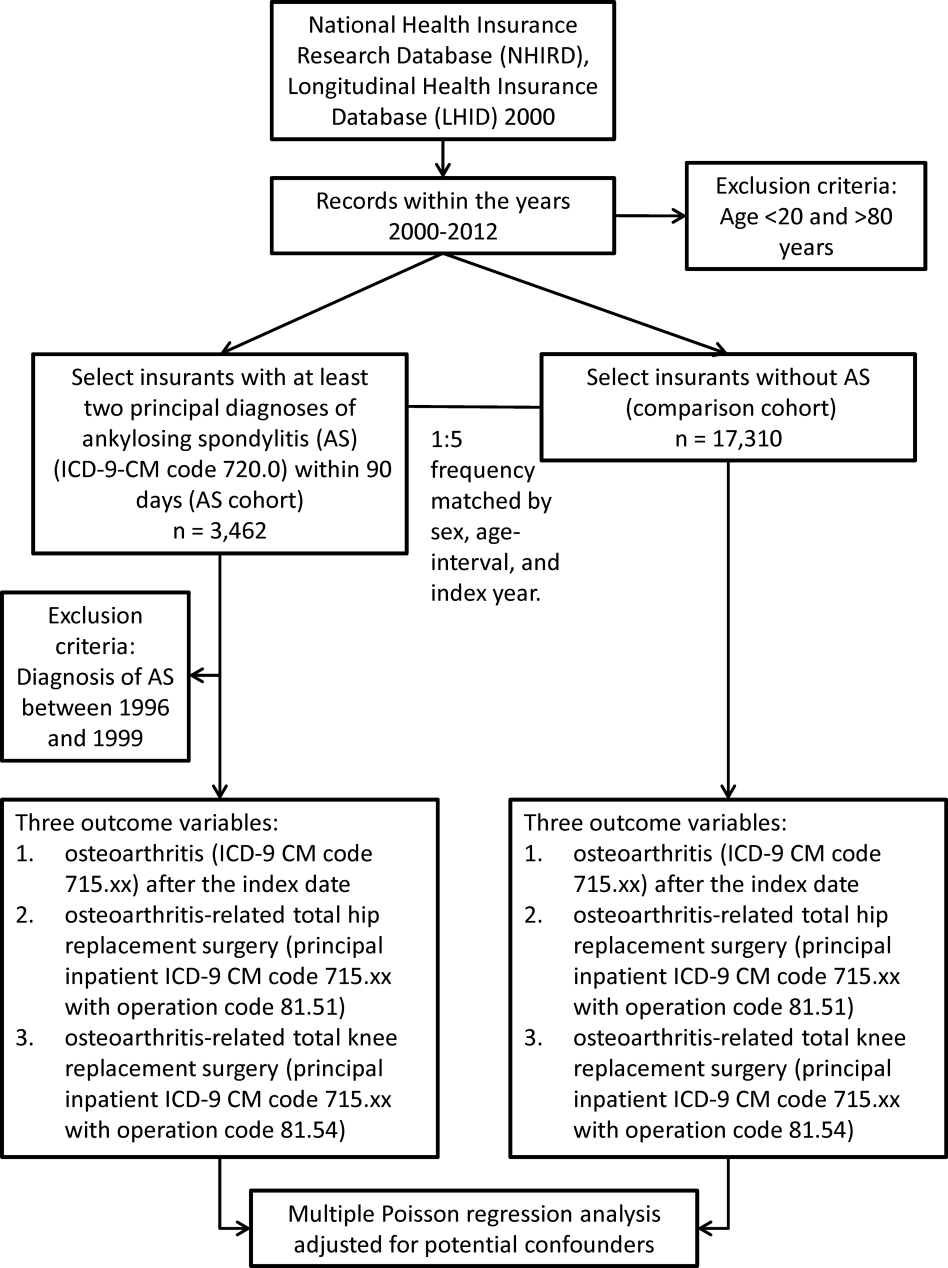
Flow diagram of the study.

A population-based, retrospective cohort study design was used to conduct a secondary data analysis based on the Longitudinal Health Insurance Database 2000 (LHID 2000), which is a subset of the Taiwan's National Health Insurance Research Database (NHIRD) (Fig 1). The LHID 2000 contained health claim data for one million beneficiaries randomly sampled from all health insurance enrollees in the Registry of Beneficiaries of the NHIRD in 2000. It represented approximately 5% of the Taiwanese population enrolled in 2000 [11]. The database was updated annually up to 2012.

The study protocol was reviewed and approved by the institutional review board of the Dalin Tzu Chi Hospital, Buddhist Tzu Chi Medical Foundation, Taiwan (No. B10502016). Since the NHIRD data files contain only de-identified secondary data, the need for informed consent from each individual patient was waived by the institutional review board.

### Identification of the ankylosing spondylitis cohort and a comparison cohort

The LHID 2000 was searched from January 1, 2000 to December 31, 2012 to assemble a cohort of patients with AS. Newly diagnosed patients, aged between 20 to 80 years, with ankylosing spondylitis were identified based on the International Classification of Diseases, 9^th^ Revision, Clinical Modification (ICD-9-CM) codes 720.0. Patients with at least two principal diagnoses of AS within a period of 90 days were considered as a definitive diagnosis of the disease in this study. Patients who had diagnosed with AS between January 1, 1996 and December 31, 1999 were excluded from the study.

The comparison cohort was assembled by randomly selected patients from the LHID 2000 with claims record between January 1, 2000 and December 31, 2012. Five patients were selected, based on frequency matching for sex, six age intervals (20.0–39, 30.0–39.9, 40.0–49.9, 50.0–59.9, 60.0–69.9, and 70.0–80.0 years), and index year with AS patients. Patients diagnosed with AS between January 1, 1996 and December 31, 1999 were excluded from the study. In Taiwan, the diagnosis of AS is generally based on the 1984 modified New York criteria [12] and more recently, the Assessment of SpondyloArthritis international Society (ASAS) classification criteria [13].

### Identification of osteoarthritis and osteoarthritis-related surgery

Both the AS cohort and the comparison cohort were followed until a diagnosis of our study outcome or the end of the follow-up period. Three study outcomes were evaluated in this study, and they included outpatient diagnosis of osteoarthritis, THRS for hip osteoarthritis, and TKRS for knee osteoarthritis. The latter two outcomes, which represented severe cases of osteoarthritis requiring surgical treatment, were included in this study to minimize surveillance bias as a result of increased medical monitoring in patients with AS[14].

In this study, a definitive diagnosis of osteoarthritis was defined as at least two diagnoses of ICD-9-CM code 715.xx within a period of 90 days. Patients with a diagnosis of osteoarthritis before the index date were excluded. For osteoarthritis-related surgery, definitive diagnoses of osteoarthritis-related THRS and TKRS were defined in this study by a principal inpatient ICD-9-CM diagnosis code of 715.xx together with ICD-9-CM procedure codes of 81.51 and 81.54, respectively. Patients with osteoarthritis-related THRS and TKRS before the index date were excluded. Fractures of the lower limb (ICD-9-CM codes 820–829) and obesity (ICD-9-CM code 278.0x) as potential confounders, were also identified from the LHID 2000.

### Statistical analysis

Sex and age intervals between the AS cohort and the comparison cohort were compared with Chi-square test and t-test, as appropriate. The incidence rate per 100,000 person-years was calculated for osteoarthritis, THRS for hip osteoarthritis, and TKRS for knee osteoarthritis separately for the AS cohort and the comparison cohort. Incidence rate ratios (IRR) for the outcome variables were calculated using Poisson regression models (i.e., generalized linear models with a Poisson log-linear link function and person-years as the offset variable), with and without adjusting for the potential confounding effect of fractures of the lower limb and obesity. Additional subgroup analyses were also performed with stratification by sex or age groups (20–39 versus 40–80 years). A two-tailed *P* value of < 0.05 was considered statistically significant. All statistical analyses were conducted using IBM SPSS Statistics software package, version 24.0 (IBM Corp, Armonk, NY, USA).

## Results

A total of 3,462 patients with AS and 17,310 patients without AS were included in this study. Table 1 shows the distribution of sex and age interval at baseline between the AS cohort and the comparison cohort. As the patients in the comparison cohort were sampled using frequency matching with those in the AS cohort, there were no significant differences between the two groups in sex and age interval. There were also no significant differences in the two potential confounding variables, obesity and fractures of the lower limb, between the two groups.

**Table 1:** Basic characteristics of the ankylosing spondylitis cohort and comparison cohort (N = 20,772).

Variable	n (%)		*P* value
	Ankylosing spondylitis cohort 3,462 (16.7)	Comparison cohort 17,310 (83.3)	
Sex			> 0.999
male	2,148 (62.0)	10,740 (62.0)	
female	1,314 (38.0)	6,570 (38.0)	
Age interval at entry (years)			> 0.999
20.0–29.9	910 (26.3)	4,550 (26.3)	
30.0–39.9	748 (21.6)	3,740 (21.6)	
40.0–49.9	701 (20.2)	3,505 (20.2)	
50.0–59.9	494 (14.3)	2,470 (14.3)	
60.0–69.9	365 (10.5)	1,825 (10.5)	
70.0–80.0	244 (7.0)	1,220 (7.0)	
Mean age (standard deviation) (years)	42.9 (15.8)	43.2 (15.8)	0.313
Median (interquartile range) (years)	40.8 (29.5–54.3)	41.1 (29.6–54.5)	
Obesity	45 (1.3)	227 (1.3)	0.935
Fractures of the lower limb	170 (4.9)	779 (4.5)	0.291

% are column percentages except in the header row where they are row percentages.

Table 2 displays the incidence rates and IRRs of osteoarthritis, THRS, and TKRS for the AS cohort and the comparison cohort. Osteoarthritis and THRS exhibited a significantly higher incidence in the AS cohort compared with the comparison cohort (adjusted IRR 1.43; *P <* 0.001 and adjusted IRR 5.91; *P <* 0.001, respectively). However, no significant differences were observed between the IRRs of TKRS between the AS cohort and the comparison cohort.

**Table 2:** Incidence rates and incidence rate ratios of osteoarthritis and osteoarthritis-related surgery for the ankylosing spondylitis cohort and comparison cohort (N = 20,772).

Disorder (ICD-9-CM)	Ankylosing spondylitis cohort			Comparison cohort			IRR (95% CI) *P* value	aIRR (95% CI) *P* value
	No. of patient	Person-years	IR	No. of patients	Person-years	IR		
**OA** (715.xx)	879	19,622	4,479.57	3,262	104,595	3,118.70	1.44 (1.33–1.55) < 0.001	1.43 (1.33–1.54) < 0.001
**OA-related surgery**								
THRS (81.51)	27	24,354	110.87	23	122,226	18.82	5.89 (3.38–10.28) < 0.001	5.91 (3.39–10.30) < 0.001
TKRS (81.54)	41	24,339	168.45	187	121,532	153.87	1.10 (0.78–1.54) 0.599	1.10 (0.78–1.54) 0.591

IR, incidence rate per 100,000 person-years; IRR, incidence rate ratio; aIRR, incidence rate ratio adjusted for obesity and fractures of the lower limb; OA, osteoarthritis; THRS, total hip replacement surgery; TKRS, total knee replacement surgery.

Table 3 showed the incidence rates and IRRs of osteoarthritis, THRS, and TKRS for the AS cohort and the comparison cohort, stratified by sex. Similar magnitudes of adjusted IRRs were observed in male patients (adjusted IRR 1.43; *P* < 0.001) and female patients (adjusted IRR 1.46; *P* < 0.001) for osteoarthritis. However, for THRS and TKRS, the IRRs were significant only among male patients (adjusted IRR 12.59; *P <* 0.001 and adjusted IRR 1.89; *P =* 0.036, respectively).

**Table 3:** Incidence rates and incidence rate ratios of osteoarthritis and osteoarthritis-related surgery for the ankylosing spondylitis cohort and comparison cohort, with stratification by sex (N = 20,772).

Outcome variable	Sex	Ankylosing spondylitis cohort			Comparison cohort			IRR (95% CI) *P* value	aIRR (95% CI) *P* value
		No. of events	Person- years	IR	No. of events	Person- years	IR		
**OA**									
	male	415	13,027	3,185.61	1,526	68,443	2,229.58	1.43 (1.28–1.59) < 0.001	1.43 (1.28–1.59) < 0.001
	female	464	6,595	7,035.54	1,736	36,152	4,801.99	1.47 (1.32–1.62) < 0.001	1.46 (1.32–1.62) < 0.001
**OA-related surgery**									
THRS									
	male	20	15,196	131.61	8	76,409	10.47	12.57 (5.54–28.54) < 0.001	12.59 (5.54–28.58) < 0.001
	female	7	9,158	76.44	15	45,817	32.74	2.34 (0.95–5.73) 0.064	2.34 (0.95–5.73) 0.064
TKRS									
	male	15	1,5260	98.29	40	76,318	52.41	1.88 (1.04–3.40) 0.038	1.89 (1.04–3.41) 0.036
	female	26	9,078	286.40	147	45,214	325.12	0.88 (0.58–1.34) 0.551	0.88 (0.59–1.34) 0.554

IR, incidence rate per 100,000 person-years; IRR, incidence rate ratio; aIRR, incidence rate ratio adjusted for obesity and fractures of the lower limb; OA, osteoarthritis; THRS, total hip replacement surgery; TKRS, total knee replacement surgery.

Table 4 showed the incidence rates and IRRs of osteoarthritis, THRS, and TKRS for the AS cohort and the comparison cohort, stratified by two age groups. The adjusted IRRs were significant for osteoarthritis in both age groups (*P* < 0.001) with similar magnitude. In contrast, the IRR for THRS in the 20–39 years group (adjusted IRR 27.66; *P* < 0.001) exhibited a large magnitude compared with THRS in the 40–80 years group (adjusted IRR 3.84; *P* < 0.001). Moreover, no patients were found to receive TKRS in the 20–39 years group and the adjusted IRRs for TKRS in the 40–80 years group was not significant.

**Table 4:** Incidence rates and incidence rate ratios of osteoarthritis and osteoarthritis-related surgery for the ankylosing spondylitis cohort and comparison cohort, with stratification by age group (N = 20,772).

Outcome variable	Age group (years)	Ankylosing spondylitis cohort			Comparison cohort			IRR (95% CI) *P* value	aIRR (95% CI) *P* value
		No. of events	Person- years	IR	No. of events	Person- years	IR		
**OA**									
	20–39	179	10,838	1,651.62	511	56,522	904.08	1.83 (1.54–2.17) < 0.001	1.82 (1.54–2.16) < 0.001
	40–80	700	8,785	7,968.49	2,751	48,073	5,722.49	1.39 (1.28–1.51) < 0.001	1.39 (1.28–1.51) < 0.001
**OA-related surgery**									
THRS									
	20–39	11	11,720	93.85	2	58,853	3.40	27.62 (6.12–124.60) < 0.001	27.66 (6.13–124.81) < 0.001
	40–80	16	12,634	126.65	21	63,373	33.14	3.82 (1.99–7.32) < 0.001	3.84 (2.00–7.36) < 0.001
TKRS									
	20–39	0	11,788	0	0	58,867	0	NA	NA
	40–80	41	12,551	326.67	187	62,664	298.41	1.10 (0.78–1.54) 0.600	1.10 (0.78–1.54) 0.589

IR, incidence rate per 100,000 person-years; IRR, incidence rate ratio; aIRR, incidence rate ratio adjusted for obesity and fractures of the lower limb; OA, osteoarthritis; THRS, total hip replacement surgery; TKRS, total knee replacement surgery; NA, not applicable.

## Discussion

In this study, we found that male patients with AS showed a significantly higher risk of osteoarthritis, TKRS, and THRS. However, in female patients with AS, only the risk of osteoarthritis was significantly higher. Our finding of an increased risk of THRS and TKRS is consistent with previous studies showed that while male patients with AS did not suffer from a higher disease activity, the radiographic damage was more severe compared with female patients [15–17]. The sexual dimorphism in AS might be explained by a difference in the in the activation status of the immune system, particularly in the inflammatory Th17 axis [18]. The increased risk of outpatient diagnosis of osteoarthritis observed in female patients with AS could plausibly be explained by surveillance bias because patients with AS are more likely to visit physicians and, therefore, have a higher chance of diagnosed with osteoarthritis compared with healthy individuals.

When the data was stratified by age group, we found that the risk of receiving THRS for osteoarthritis was significantly higher in patients with AS who were under 40 years of age compared with those who were older. We speculated that while hip destruction in patients with AS is a direct involvement of the pathogenesis of AS, damage in the knee joint is probably synergistic with the degenerative change and disease activity of AS. The very high risk of THRS among young patients with AS (adjusted IRR 27.66; *P* < 0.001) is a notable finding in this study. Because of limited longevity of joint implants, a revision hip replacement in young recipients is expected during their lifetime, but previous studies of young patients with AS have shown that THRS performed on them was associated with high complication rates [19,20]. Currently, biologic agents, especially anti-tumor necrosis factor agents are used for treating patients with AS [21]. AS patients with the presence of peripheral arthritis have been reported to respond well to biologic agents [22,23]. Future studies should investigate whether biologic agents could defer the need for THRS in patients with AS.

Several limitations should be noted when interpreting the results from this study. First, the identification of AS, osteoarthritis, THRS, and TKRS were based solely on ICD-9-CM codes. Nevertheless, the National Health Insurance Administration routinely performs audits on random samples of medical claims to ensure their accuracy. Second, no information is available on the severity of AS. Despite these caveats, the strengths of this study included its population-based cohort design and a long duration of follow-up. Furthermore, the evaluation of osteoarthritis-related THRS and TKRS, in addition to outpatient diagnosis of osteoarthritis, provided results that are less likely to be affected by potential misclassification bias arising from heightened medical surveillance among patients with AS.

In conclusion, this secondary cohort analysis of a nationwide, population-based health claims database showed that male patients with AS had a significantly higher risk of developing osteoarthritis and receiving osteoarthritis-related THRS and TKRS. A high risk of receiving THRS among young patients with AS deserves special attention because revision THRS in these patients are known to be associated with high complication rates.
